# Whole-exome Sequencing of Prostate Cancer in Sardinian Identify Recurrent UDP-glucuronosyltransferase Amplifications

**DOI:** 10.7150/jca.48433

**Published:** 2021-01-01

**Authors:** Tiansheng Zeng, Maria Antonietta Fedeli, Francesco Tanda, Yuyong Wang, Dongsheng Yang, Bei Xue, Lisha Jia, Giuseppe Palmieri, Leonardo A Sechi, David J. Kelvin

**Affiliations:** 1 Division of Immunology, International Institute of Infection and Immunity, Shantou University Medical College, Shantou Guangdong, China.; 2 Department of Biomedical Sciences, University of Sassari, Sassari, Italy.; 3 Department of Scienze Mediche Chirurgiche e Sperimentali, first affiliated Hospital of 33445Sassari University.; 4 Department of Urology, affiliated Hangzhou First People's Hospital, Zhejiang University School of Medicine, China.; 5 Institute of Genetic and Biomedical Research (IRGB), Head, National Research Council (CNR), 07100 Sassari, Italy.; 6 Department of Microbiology and Immunology, Dalhousie University, Halifax, Nova Scotia, Canada B3H 4R2.; 7 Canadian Center for Vaccinology, IWK, Halifax, Nova Scotia, Canada.

**Keywords:** UDP glucuronosyltransferase, BTBD7-SLC2A5 fusion, prostate cancer, Sardinian, whole exome sequencing

## Abstract

Globally, prostate cancer is the third most common cancer in the world, and the second most common cancer in men. However, rates for incidence and mortality vary considerably with race, ethnicity, and geography. Over 97 significantly mutated genes that have been identified in prostate cancer; however, a lack of genomic prostate cancer studies focusing on different racial and ethnic groups and racial mixing pose a serious challenge to universalize these findings. The Sardinian population is an isolated Mediterranean population that has a high frequency of centenarians and a much lower incidence of prostate cancer than found in males in mainland Europe. Here, we conducted a genomic prostate cancer study on a Sardinian cohort diagnosed with local prostate cancer. Our data reveals a low rate of ERG fusion in Sardinian prostate cancer. Interestingly, we identified a novel BTBD7-SLC2A5 fusion that occurred in 13% of the patients. We also found that the UGT2B4 on 4q13.2 was amplified in 20% of the Sardinian patients but rarely amplified in patients of other population. These observations underscore the importance of the inter-population molecular heterogeneity of prostate cancer. In addition, we examined the expression of UGT2B4 in 497 prostate cancer patients derived from The Cancer Genome Atlas database. We found that high expression of UGT2B4 was associated with low-grade prostate cancer and upregulation of UGT2B4 in tumors was associated with upregulation of metabolism pathways such as 'de novo' IMP biosynthetic process, glutamine and monocarboxylic acid metabolism. These data provide insight into clinical relevance and functional mechanism of UGT2B4. Further understanding functional mechanism of UGT2B4 amplification and BTBD7-SLC2A5 fusion will aid in developing drugs to benefit the prostate cancer patients.

## Introduction

Globally, prostate cancer is the third most common cancer in the world, and the second most common cancer in men 1. Prostate cancer rates for incidence and mortality vary considerably with race, ethnicity, and geography 2. However, other factors such as socioeconomic status and access to health care create differences in the diagnosis, treatment, and survival of prostate cancer patients. Intriguingly, genetic factors are a likely major cause for differences in the molecular basis, incidence, and mortality of prostate cancer 3-7.

Genome-wide association studies have identified over 100 low penetrance germline genomic loci associated with prostate cancer risk. These low penetrance loci may account for about 30% of the risk 8, 9. Germline variants of genes with high penetrance mutations for prostate cancer susceptibility, including HOXB13, BRCA1/2, and DNA mismatch repair genes, account for about another 5% of prostate cancer risk 10, 11. Somatic mutations in prostate cancer-driver genes play a significant role in tumorigenesis and development, and mutations in over 97 genes have been identified that act as cancer driver genes, including mutations for TMPRSS2-ERG, TP53, and PTEN 12. Somatic and germline mutations are under investigation as both therapeutic targets and biomarkers of risk and outcome 12-14. A lack of genomic prostate cancer studies focusing on different racial and ethnic groups and racial mixing poses a serious challenge to a comprehensive understanding of genetic risk, diagnosis, and treatment of prostate cancer patients 15. Additionally, few studies have focused on isolated regional populations. Sardinia is an island in the western Mediterranean Sea, with the European mainland to the north and North Africa to the south. The population of Sardinia has long been a focus of genetic and genomic studies on ageing and autoimmunity as there is a high incidence of centenarians as well as Type I diabetes and multiple sclerosis 16-20. However, the incidence of prostate cancer in Sardinia (44/100,000) appears to be lower than that in mainland Europe (approximately 100/100,000 in North Europe) 2, 21. Furthermore, Chiang et al. demonstrated that the Sardinian population is an isolated Mediterranean population, with the evolutionary divergence from the European mainland population taking place some 143.3±1.3 generations (about 4,300 years ago) 22. In this study, we performed whole exome sequencing on tumor and tumor-adjacent tissues from 30 local prostate cancer patients in Sardinia to investigate the effects of genetic factors on prostate cancer in the Sardinian population.

## Materials and Methods

### Sardinian patients and samples

This research was approved by the Ethical Committee of the Sassari AOU. Our study included 30 patients who diagnosis as prostate cancer and received surgery in 2010. Patients with localized prostate cancer that had prostate resection before ADT treatment were inclusion in this study. Patients with metastatic disease or received ADT treatment before tumor resection were exclusive. Most patients had levels of PSA <10 ng/ml and Gleason scores ≤7 (low risk, 23 out of 30 patients). Six patients had PSA levels between 10 ng/mg and 20 ng/ml and Gleason scores ≤7 (intermediate risk, 6 out of 30), and one patient had a Gleason score of 9 (high risk, 1 out of 30). The age distribution of the patients ranged from 54 to 74. In these patients, twenty were 60~69 years old, four were 53 to 59 years old, and six were 70 to 74 years old. Survival records showed that one patient died within one year after diagnosis, while another four patients deceased 5 years after diagnosis. All patients had prostate resection before ADT treatment. Clinical information is summarized in** Table 1**.

Tissue was obtained from tumor resections from patients who received radical prostatectomy and were formalin-fixed paraffin-embedded and stained with hematoxylin and eosin. All the Formalin-fixed paraffin-embedded tissue (FFPET) were obtained from the Pathology Section of the Department of Experimental Medicine of the University of Sassari (Sassari, Sardinia, Italy). Paraffin blocks were marked boundaries between tumor lesions and corresponding tumor adjacent tissues and then cut into two sections based on boundaries. In principle, the tumor lesions were engaged into sequencing as tumor tissue, the corresponding tumor adjacent tissues were sequenced as controls.

### The Cancer Genome Atlas (TCGA) patients

From the Cancer Genome Atlas (TCGA) database (https://cancergenome.nih.gov/), a total of 497 primary prostate cancer patients with gene expression profiles were identified and used in this study. Clinical characteristics at time of diagnosis, including PSA levels, Gleason score, and clinical stage were described. Disease-free survival (DFS) was the primary end point of this study. DFS was defined as the time from diagnosis to the first event, including biochemical recurrence, death, on the last follow-up. Between-group comparisons of DFS were performed by the Kaplan-Meier method and the log-rank test. Gene differential expression of TCGA pan-cancer was performed by TIMER DiffExp module 23.

### DNA and RNA extraction

DNA was extracted from 250 ul of whole peripheral blood by QIAamp DNA Blood Mini Kit with a total amount of 10-50 ug. The QIAGEN GeneRead DNA FFPE Kit, which enables purification of high-quality genomic DNA and removes artificial C>T mutations, was used to extract DNA from the formalin-fixed paraffin-embedded tissue (FFPET). RNA extraction from FFPET was performed using a QIAGEN RNeasy FFPE Kit that was specifically designed for purifying total RNA from formalin-fixed, paraffin-embedded tissue sections. All the above procedures were performed following the manufacturer's protocols. The concentration and quality of the extracted DNA and RNA were determined by Qubit3.0 and Agilent 2100. The qualified DNA that with the fragment size more than 800 bp was engaged into the next library step. RNA was reversed transcript to cDNA for ETS fusion detection.

### ERG fusion detection by polymerase chain reaction (PCR)

ERG fusion detections were performed by polymerase chain reaction PCR. cDNA was generated using SuperScript IV One-Step RT-PCR System (Applied Biosystems TM, USA). PCR was performed with the primers F: GCTCCTATCACGCCCACCCA and R: TCCTTCCCCAGCCCCAGTAAA to estimate the expression of the ERG gene. As contrast, the expression of the housekeeping gene glyceraldehyde-3-phosphate dehydrogenase were detected with primers F: TGCACCACCAACTGCTTAGC, R: GGCATGGACTGTGGTCATGAG. Samples with ERG expression were selected to determine if any samples contained any of the known 5 types of breakpoints for TMPRSS2-ERG fusions. The primers for detection of each breakpoint are listed in **Supplementary Table S1**.

### Whole exome sequencing library preparation

100-200 ng DNA per sample was used for library construction using the KAPA Hyper-Plus Kit. Enzymatic fragmentation was performed according to the manufacturer's instructions. Fragments sized 180-220 bp before adapter ligation were selected for capture using the Roche Seq-Cap EZ Med-Exome system resulting in a total capture of 67Mb. Libraries were analyzed for size distribution by Agilent 2100 Bioanalyzer and quantified by real-time PCR. The qualified libraries were sequenced by the Illumina X-ten platform with 8 samples per lane.

### Sequence data quality control

The original fluorescence image files obtained from the X-ten platform were transformed to short reads (raw data) by base calling and the short reads were saved in FASTQ format, containing sequence information and corresponding sequencing quality information. Reads were filtered as follows: 1) Discard paired reads if either one read contained adapter contamination (>10 nucleotides aligned to the adapter, allowing ≤ 10% mismatches); 2) Discard paired reads if more than 10% of bases were uncertain in either one read; 3) Discard paired reads if the proportion of low quality (Phred quality <5) bases was over 50% in either one read. All the downstream bioinformatic analyses were performed on high-quality clean data.

### Read mapping and processing

Sequencing data was mapped to the reference human genome (UCSC hg19) by Burrows-Wheeler Aligner (BWA) software to get the original mapping results in BAM format 24. Then SAMtools, Picard, and GATK tool kits were used to sort BAM files and duplicate marking, local realignment, and base quality recalibration to generate a final BAM file for somatic and germline SNVs and indels calling 25, 26.

### Somatic SNP and INDEL calling and annotation

Somatic SNVs were identified by GATK muTect1. Somatic indels were identified by Pindel based on paired bam files that were generated from paired tumor and tumor-adjacent tissues 27. The minimal depth for high confidence SNVs was set as 10, while a depth of 20 was set for indels. Based on high sensitivity of the Pindel algorithm and the possible damage of DNA due to historical FFPETs (>5 years), single nucleotide deletions were removed from the final results. Annotation of the somatic SNVs and indels was by performed using Oncotator 28. The annotated MAF files were used for downstream analysis.

### Fusion calling, filtering, ORF prediction, and visualization

Fusion event calling from raw read files of tumor-adjacent tissues and tumor lesions was performed using FusionMap 29 which was designed to detect and align fusion junction-spanning reads to the genome directly 29. Fusions were filtered out if 1) seed reads were ≤3 or were listed in the in-family analysis or found in a paralog gene list; 2) fusions were associated with uncharacterized genes, immunoglobin genes, mitochondrial genes or repeat regions; and 3) fusions were reported in normal samples. High confidence fusions were input into FusionHub to search for reported fusions and predict the fusion effects.

### Copy number variation calling, filtering, and driver copy number variation identification

Control-FREEC was used to detect CNV with paired pileup files that were generated from unsorted bam files 30. High confidence somatic CNVs were identified with a *p*-value less than 0.01 for both Wilcoxon and the Kolmogorov-Smirnov tests. They were next annotated by ANNOVAR 31. Intergenic regions were then removed. Copy number variation reoccurring analysis was performed using GISTIC2.0 32.

Sardinian prostate cancer driver copy number variations already present in OncoKB^33^ (https://www.oncokb.org) annotated alterations were classified as “putative copy number variations”. Classification of Sardinian “candidate driver copy number variations” was based on curation in OncoKB onco-gene (amplification) or in OncoKB tumor suppressor gene (loss).

### Functional enrichment and protein-protein interaction network analysis

GO, KEGG, and Reactome pathway enrichment analyses of a given gene set were performed by metascape 34. Protein-protein interaction network of a given gene set was obtained from InWeb_IM 35 and visualized by Cytoscape 36.

### Identification of novel driver

Candidate prostate cancer driver analysis with the whole exome data of 30 patients with tumor adjacent and tumor samples were conducted by OncodriverCluster 37 then annotated with MutationAssessor 38, SIFT 39, Polyphen2 40, and FATHMM 41. Mutations that showed significance in OncodriverCluster analysis (*p* value <0.01) and annotated as damage mutations by at least three of the four methods MutationAssessor, SIFT, Polyphen2, and FATHMM were retained. The remaining mutations were mapped to OncoKB database 33 (www.oncokb.org/cancerAlterations). Mutations that have not been recorded as putative drivers in Oncokb database were retained as novel driver candidates.

## Results

### Genomic landscape of Sardinian prostate cancer

**Figure 1:**
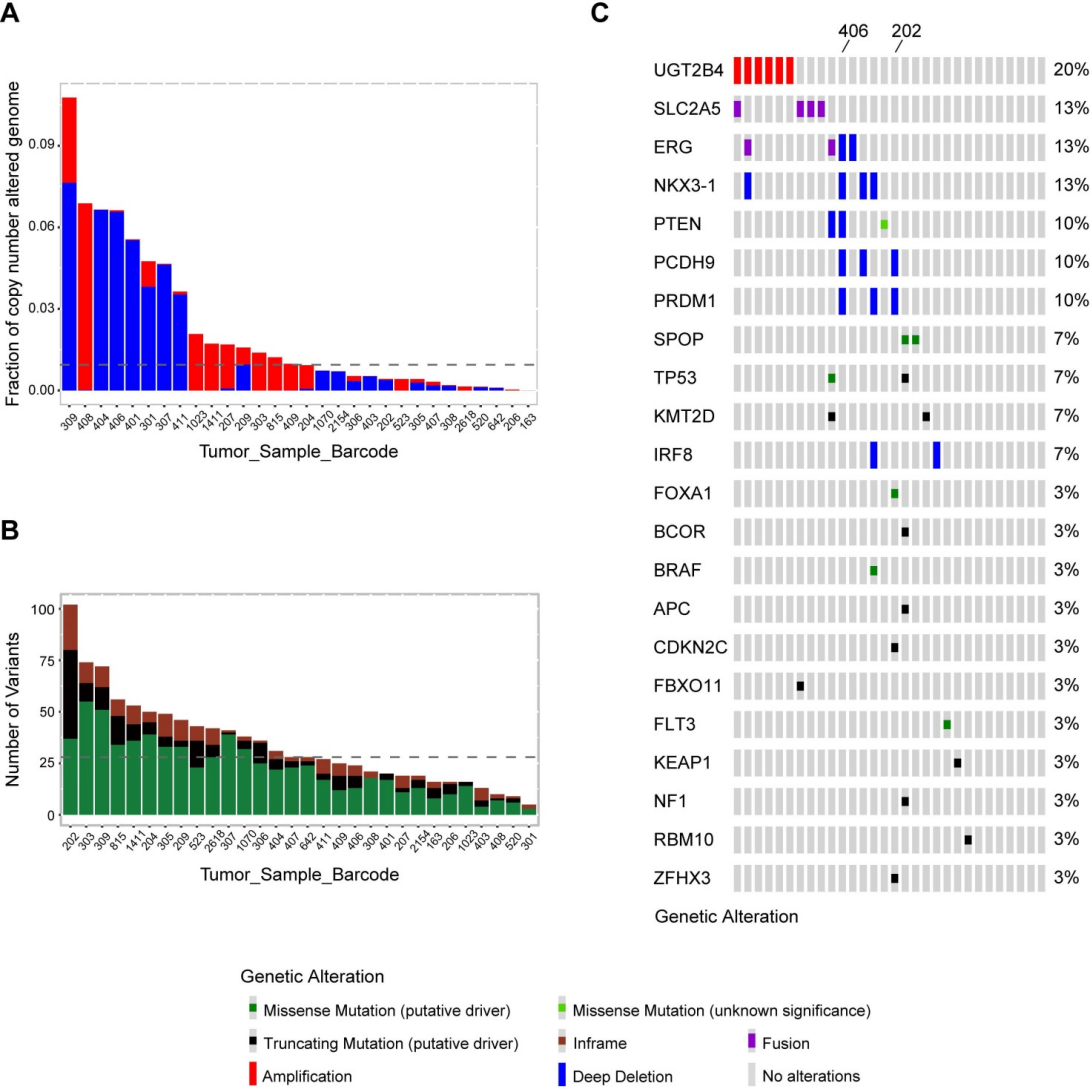
** Genomic landscape of Sardinian prostate cancer. A)** Fraction of genomic copy number alterations in 30 Sardinian local prostate cancers determined by whole exome sequencing of paired tumor and tumor-adjacent tissue. Amplifications are highlighted by red; deep deletions are highlighted by blue. The highest fraction of altered genome was observed in patient 309, at 0.107. Patient 163 had the lowest fraction of altered genome at 0.00001. The median was 0.009. **B)** Number of somatic nonsynonymous mutations in 30 Sardinian local prostate cancer samples determined by whole exome sequencing of paired tumor and tumor-adjacent tissues. Truncating mutations are highlighted in black; missense mutations are highlighted in green; the in-frame indels are highlighted in brown. Patient 202 had the highest number of somatic non-synonymous mutations, 103. Patient 301 had the lowest number of non-synonymous mutations, 7. The median is 28 non-synonymous mutations per patient. **C)** Oncoplot of comprehensive molecular profiling of 30 Sardinian local prostate cancer samples were obtained by whole exome sequencing of paired tumor and tumor-adjacent tissues. Each red bar represents a patient with amplification of the specified gene on the left. Each blue bar represents a patient with deep deletion of the specified gene. Each green dot represents a patient with a putative missense mutation; black dot represents a patient with putative truncating mutation; purple dot represents a patient with fusion of the specified gene. UGT2B4 was amplified in 20% of the tumors and the novel BTBD7-SLC2A5 fusion was observed in 13% of the tumors. Recurrent alterations of putative drivers in prostate cancer, such as ERG, NKX3-1 PTEN, SPOP, FOXA1, were also observed in Sardinian patients with local prostate cancer. Patient 202 had the largest number of somatic putative mutations and patient 406 had the largest number of tumor suppression genes deleted.

Whole exome sequencing was conducted in tumor and matched tumor-adjacent FFPETs sections from 30 Sardinian prostate cancer patients. Sequencing coverage of tumor tissues was 39-176, with the median of 67. The sequencing coverage of histologically normal tissue adjacent to tumor tissue was 30-156, and the median was 58. The fraction of copy number alterations genome across 30 Sardinian local prostate cancer samples was 0.107~0.00001 with the median at 0.009. Patient 309 had the highest fraction of copy number variations and Patient 163 had the lowest** (Figure 1A).** The number of nonsynonymous mutations of each of the 30 Sardinian local prostate cancer patients was 7-103, with a median of 28 mutations per patient. Patient 202 had the most somatic nonsynonymous mutations and patient 301 had the lowest number of mutations **(Figure 1B).**

Comprehensive molecular profiling including somatic mutations, copy number variations, and fusion events were characterized by whole exome sequencing data for 30 Sardinian local prostate cancer patients. Our data showed that frequently observed oncogenic drivers in prostate cancer tumors found in previous studies 13, 42, such as mutations in SPOP, KMT2D, TP53, BRAF, FLT3, APC, BCOR, CDKN2C, FBXO11, KEAP1, FBXO11, NF1, RBM10 and ZFHX3, were also observed in Sardinian prostate cancer tumors **(Figure 1C).** Most of the oncogenic drivers were observed only once, with the exception of mutations in SPOP, KMT2D and TP53, which were observed twice **(Figure 1C).** As well, frequently observed deletions of tumor suppression genes, such as NKX3-1, PTEN, PCDH9, PRDM1 and IRF8, were also observed in Sardinian prostate cancer patients **(Figure 1C).** To define the frequency of TMPRSS2-ERG fusions, a PCR-based method was employed. We examined 19 tumor samples from Sardinian patients in which RNA was available (historical FFPET samples) for possible TMPRSS2-ERG fusions. Of the 19 samples, two samples were TMPRSS2-ERG fusion positive (11%). In addition to the prostate cancer genomic signatures noted above, we found that the 4q13.2 UDP glucuronosyltransferase family was amplified in 20% of Sardinian prostate cancers. We also observed that novel fusions between the fructose transporter gene SLC2A5 and BTBD7 (Broad-Complex, Tramtrack and Bric a brac domain containing 7), occurred in 13% of the Sardinian patients** (Figure 1C).**

### Identification of 4q13.2 UDP glucuronosyltransferase family amplification in Sardinian prostate cancers

**Figure 2:**
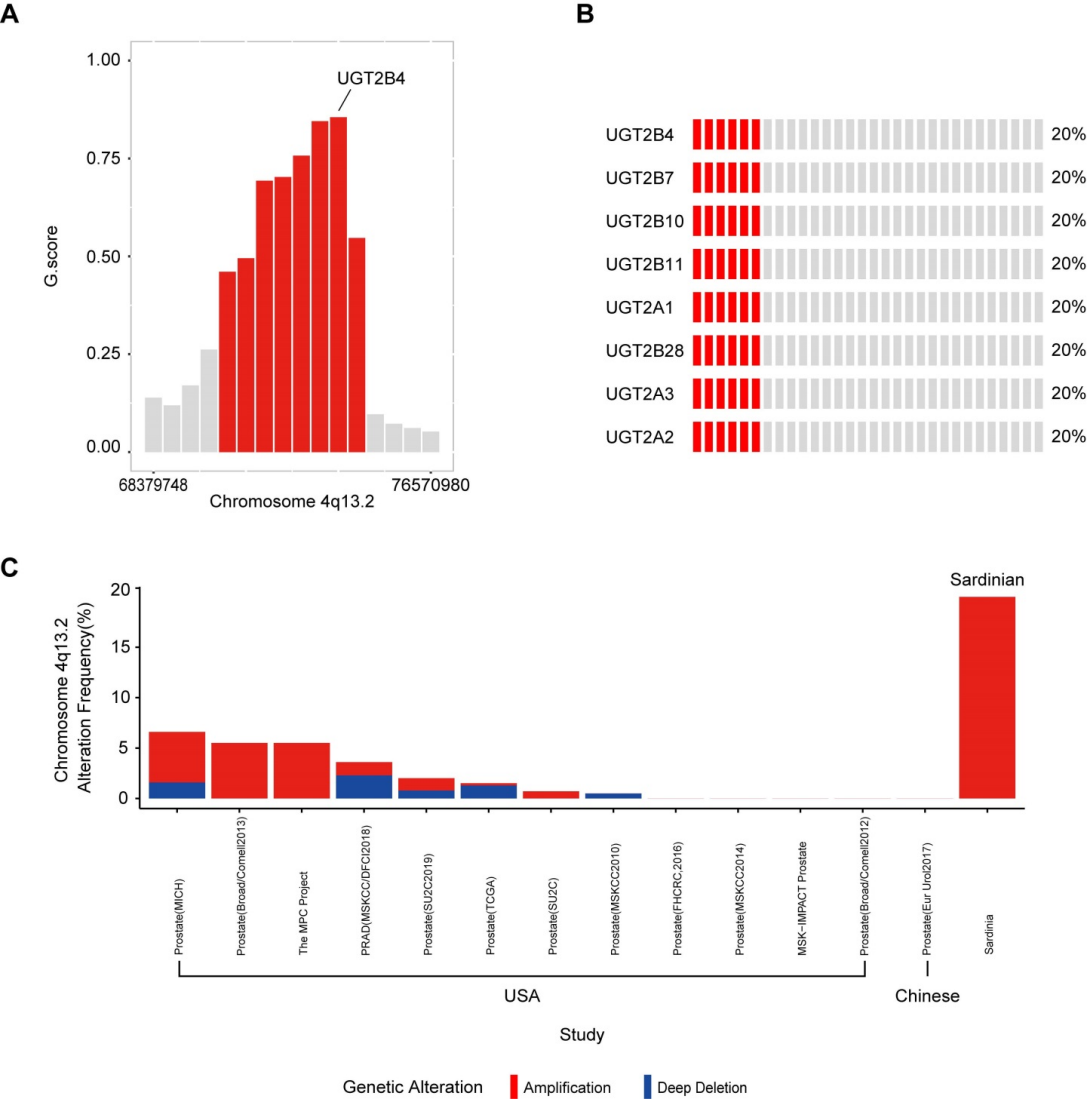
** Amplification of UGT family genes in 4q13.2. A)** The G-score was calculated using GISTIC algorithm. Chromosome 4q13.2 amplification (red) with FDR ≤0.01 was plotted. UGT2B4 was located in the peak of the 4q13.2 amplification region. The other red bars are UGT2B10, UGT2A3, UGT2B7, UGT2B11, UGT2B28, UGT2A1/UGT2A2. **B)** Oncoplot of amplifications of genes in Chromosome 4q13.2 across 30 Sardinian prostate cancer patients. Each red bar represents a patient with an amplification of the specified gene on the left. Gray bars represent patients without amplification. 20% of the Sardinian patients showed UGT family amplifications of 8 UGT genes found within the UGT2 locus.

To determine significantly amplified or deleted chromosome regions in our Sardinian prostate cancer samples, the GISTIC algorithm was used to calculate the G-score, which determines the amplitude and the frequency of copy number variations** (Supplementary Figure S1)**. The results showed that a region in chromosome 4q13.2 which contains multiple **UDP glucuronosyltransferase family** genes was amplified in 20% of Sardinian prostate cancers and the amplified peak was centered on UGT2B4. The other UGT family member amplifications included UGT2B7, UGT2B10, UGT2B11, UGT2A1, UGT2A2, UGT2B28, and UGT2A3** (Figure 2B and Figure 2C)**. The UGT family amplification we observed in Sardinian prostate cancers has only rarely been observed in 13 other prostate landscape studies 6, 12, 13, 42-46. The UGT family was amplified in 0-5.5% of prostate cancer patients in cohorts from the USA 13, 42-46 and no amplifications were observed in a cohort of Chinese patients 6 **(Figure 2C)**. We have made comparison between Sardinian patients and these 13 prostate cancer genomic studies of other race and ethnics. The UGT family amplification we observed significantly with higher rate in Sardinia prostate cancers comparing to prostate cancer in 10 prostate landscape studies as PRAD (MSKCC/DFCI2018), Prostate (SU2C2019), Prostate (TCGA), Prostate (SU2C), Prostate (MSKCC2010), Prostate (FHCRC,2016), Prostate (MSKCC2014), MSK-IMPACT Prostate, Prostate (Broad/Comell2012), Prostate (Eur Urol2017) (fisher test, *p* value <0.001). These studies were based on primary or metastatic prostate cancer. The details listed in **Supplementary Table S2**.

### Identification of BTBD7-SLC2A5 fusions in Sardinian prostate cancers

**Figure 3:**
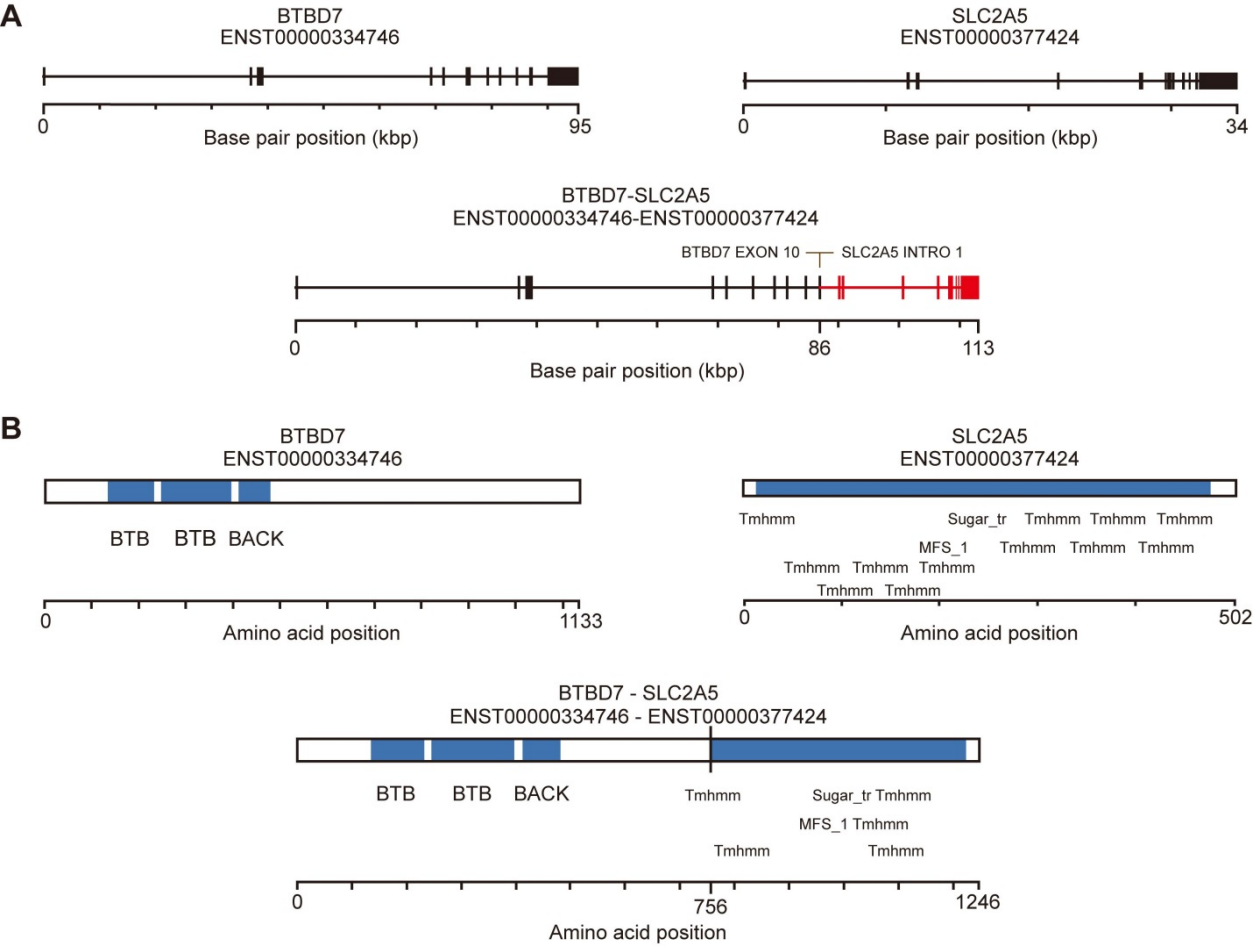
** Novel fusion BTBD7-SLC2A5 identification. A)** Line schematic of exon-intron structure of the BTBD7 and SLC2A5 genes, and the BTBD7-SLC2A5 gene fusion. All of the BTBD7-SLC2A5 fusions identified in the four patients had the same breakpoints (See Table 2 for details). The breakpoints were within exon 10 of the BTBD4 and intron 1 of SLC2A5. **B)** Predicted transcript and protein of the BTBD7-SLC2A5 fusion determined by Fusion hub. The predicted protein contained the entire transmembrane domain and fructose transporter domain of SLC2A5 and the BTB domain of BTBD7.

To identify fusion events from the whole exome data, we used FusionMap 29 to measure the junction-spanning reads from the raw reads of Sardinian prostate cancer tumor samples and tumor-adjacent samples. We identified 15, 12, 15, and 12 reads from tumor samples 306, 409, 206 and 815, in which at least 25 nts mapped in the genes for both BTBD7 and SLC2A5 **(Table 2)**. No split reads were found in the tumor-adjacent tissues. The unique cutting positions of the seed reads in the four tumor samples were 5, 6, 14, and 10 respectively. It is expected that a real fusion would have more than one unique cutting position** (Table 2)**. The breakpoints were identified within exon 10 of the BTBD7 gene and intron 1 of the SLC2A5 gene** (Figure 3A).** We used the fusion hub to predict the transcript and protein of the BTBD7-SLC2A5 fusion gene. The predicted protein contains the entire transmembrane domain and fructose transport domain of SLC2A5 and the BTB domain (Broad-Complex, Tramtrack and Bric a brac domain) of BTBD7 **(Figure 3B).**

### Identification of 9 novel somatic mutation driver candidates in Sardinian prostate cancers

**Figure 4:**
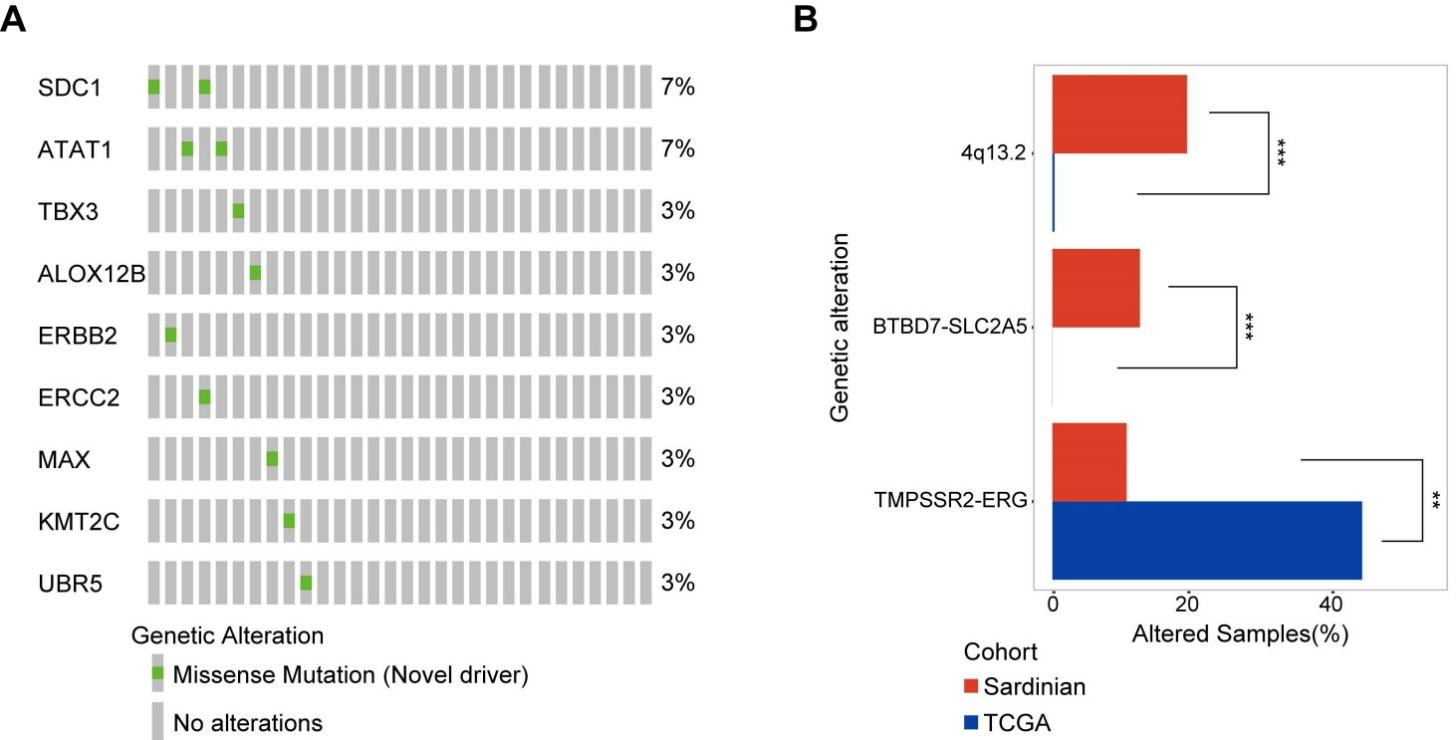
** Novel candidate driver mutations in Sardinian prostate cancer and differentially mutated genes. A)** The frequency of nine novel somatic mutations identified using OncoDriveCluster and MutationAssessor, SIFT, Polyphen2, and FATHMM are shown for each patient. The SDC1 protein with the mutation R277S, and the ATAT1 protein with the mutation D19V mutation, were observed in two patients respectively. All of the remaining mutations were observed only in a single patient each. Six of the 9 mutations have known 3D structures. **B)** Differentially mutated genes between Sardinian and The Cancer Genome Atlas prostate cancer cohorts (two side fisher test, *p* value < 0.05).

To identify novel somatic mutation candidate drivers in Sardinian prostate cancers, we firstly used OncodriverCluster 37 to predict the candidate prostate cancer mutation driver with the whole exome data of 30 patients with tumor adjacent and tumor samples. Mutations that showed significance in OncodriverCluster analysis (*p* value <0.01) and annotated as damage mutations by at least three of the four methods: MutationAssessor 38, SIFT 39, Polyphen2 40, and FATHMM 41, and have not been recorded as putative drivers in Oncokb database (www.oncokb.org) were retained. Nine novel candidate drivers were identified in prostate cancer of Sardinia, including mutations in the SDC1 protein (R277S) and the ATAT1 protein (D19V) observed in two of the samples for each protein **(Figure 4A)**. The other mutations were observed only in one sample each **(Figure 4A)**. Six of the 9 mutations have known 3D structures. They include, the ALOX12B protein (R422W, **Supplementary Figure S2A)**, the ATAT1 protein (D19V mutation**, Supplementary Figure S2B)**, the ERBB2 Receptor L domain harboring **(**C53R mutation**, Supplementary Figure S2C)**, the ERCC2 protein **(**R497C mutation**, Supplementary Figure S2D)**, the MAX protein **(**D87N mutation**, Supplementary Figure S2E)**, and the TBX3 T-box domain **(**V202D mutation**, Supplementary Figure S2F)**.

### Differentially mutated genes between Sardinian patients and The Cancer Genome Atlas prostate cancer cohort

We conducted a systematic comparison of our cohort of 30 Sardinian cancer prostate patients and 497 The Cancer Genome Atlas prostate cancers 42 to identify differentially mutated genes. Three differentially mutated genes were observed between our Sardinian cohort in this study and The Cancer Genome Atlas prostate cancer cohort (*p*<0.01 Fisher's exact test). ERG fusion was significantly less frequently altered in Sardinian prostate cancer (*p*<0.001 Fisher's exact test). Chromosome 4q13.2 amplification and BTBD7-SLC2A5 fusion were significantly more frequently altered in our Sardinian cohort (*p*<0.001 Fisher's exact test) **(Figure 4B)**.

### Clinical relevance and functional mechanism of UGT2B4 expression in prostate cancer

**Figure 5:**
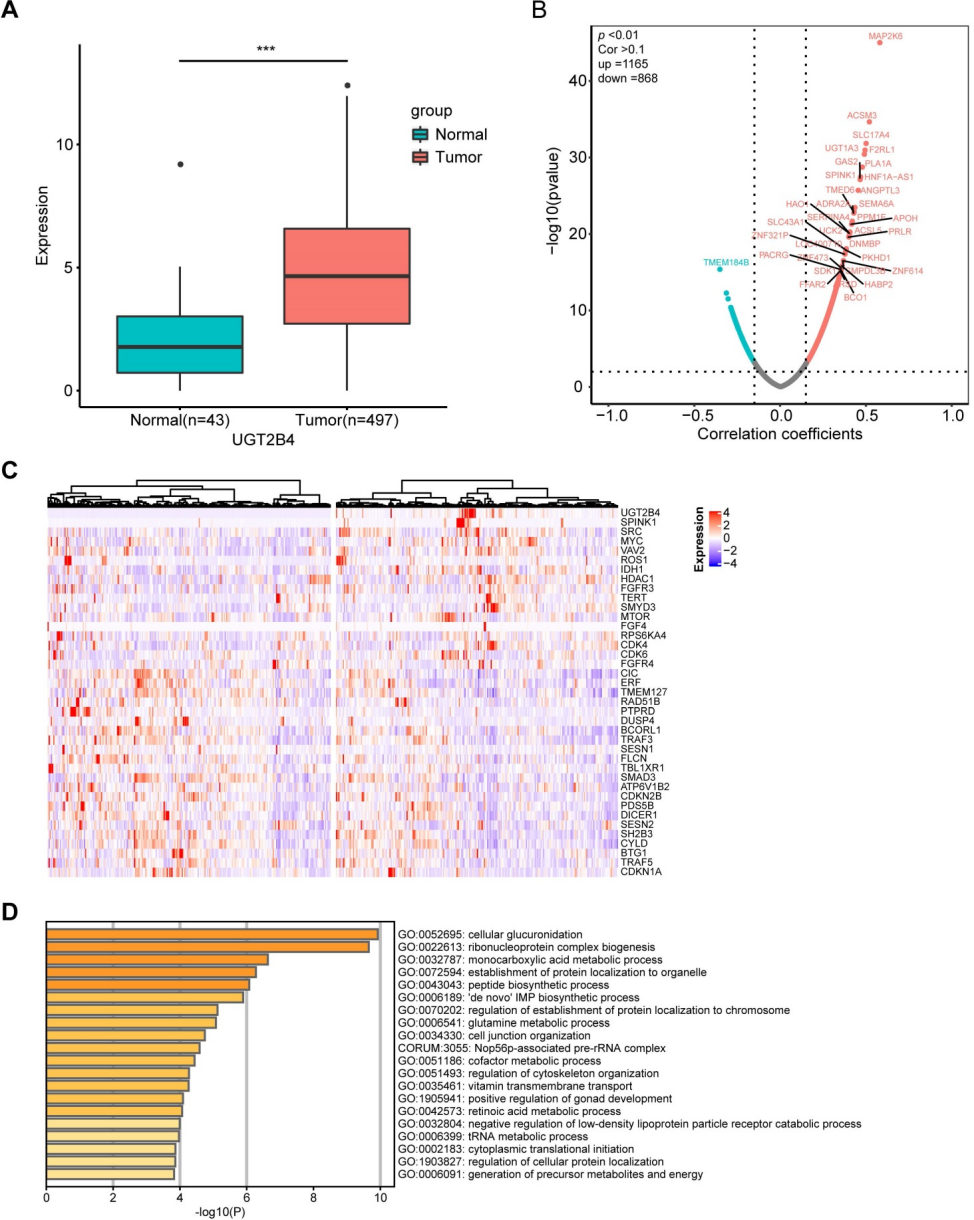
** Clinical and functional relevance of UGT2B4 expression in prostate cancer. A)** Expression of UGT2B4 was determined in 43 normal tissue (green bar) and 497 prostate cancers (red bar) from The Cancer Genome Atlas database. UGT2B4 was found to be significantly upregulated (*p*<0.001) in localized prostate tumors compared with tumor-adjacent normal tissue. **B)** Spearman rank correlation analysis was performed on expression profiles of tumor tissues of 497 TCGA localized prostate cancer patients and UGT2B4. 1165 genes were positively co-expressed with UGT2B4 and 868 genes were negatively co-expressed with UGT2B4 i. (Spearman correlation >0.1, *p*<0.01). **C)** Thirty-seven curated oncogenes and tumor suppressor genes were correlated with UGT2B4 in the spearman rank correlation analysis. Heatmap of hierarchal clustering of patients based on expression of UGT2B4 and 37 UGT2B4 co-expressed oncogenes and tumor suppressor genes was described using a function Heatmap in ComplexHeatmap R package. Patients were split by median expression of UGT2B4 before hierarchal clustering. There was a cluster of prostate cancer patients with high expression of UGT2B4, SPINK1, and SRC. **D)** Functional enrichment including GO Biological Processes, KEGG, and Reactome pathways (http://www.metascape.com/) reveals that genes co-expressed with UGT2B4 in the Spearman correlation analysis were functionally enriched in ribonucleoprotein complex biogenesis, as well as glutamine, nucleotide, and monocarboxylic acid metabolic pathways.

UGT2B4 was identified as the peak of amplification in the UGT family in our present study. To examine the functional mechanism of UGT2B4 expression in prostate cancer, we investigated the expression data of a pan-cancer study from TCGA. Our analysis showed that the expression level of UGT2B4 in breast cancer tumors was significantly lower than that in normal tissues, while UGT2B4 was significantly higher in tumors than in normal prostate cancer tissues **(Figure 5A and Supplementary Figure S3)**.

To examine the clinical relevance of UGT2B4 in prostate cancer, we downloaded gene expression profiles and clinical profiles of 497 tumors from primary prostate cancer patients in The Cancer Genome Atlas (TCGA) database. All patients were divided into UGT2B4-high and UGT2B4-low groups by the median UGT2B4 expression level of the cohort **(Supplementary Figure S4A)**. Kaplan-Meier analysis demonstrated that patients with high UGT2B4 expressers had longer disease-free survival than patients with low expressers (*p*<0.05, **(Supplementary Figure S4B)**. In addition, UGT2B4 high expressers are significantly associated with low Gleason scores (64% of patients with Gleason 6-7 in UGT2B4-high group, 54% of patients with Gleason 6-7 in UGT2B4-low group, *p*<0.05 Fisher's exact test).

Further to examine the potential functional mechanism of UGT2B4 in prostate cancer, we performed the spearman correlation analysis of UGT2B4 in the expression data of the 497 prostate cancer patients from TCGA **(Figure 5B)**. We found that the expression of UGT2B4 was positively associated with expression of multiple well-known oncogenes such as SPINK1, IDH1, MYC, and SRC and negatively associated with expression of well-known tumor suppressors such as CIC and ERF. **(Figure 5C)** Functional enrichment analysis reveals that UGT2B4 co-expression genes enriched in tumor metabolic pathways such as 'de novo' IMP biosynthetic process, glutamine and monocarboxylic acid metabolic **(Figure 5D)**. Protein interaction network of UGT2B4 co-expressed genes showed that UGT2B4 positive co-expression genes that broadly interact with SRC and MYC **(Supplementary Figure S5).**

## Discussion

Emerging evidence indicates that there are remarkable disparities in prostate cancer epidemiology as well as in the molecular landscape among different population groups of European, North American, African, and Asian origin 2, 15. For example, ETS fusion affects ~50% of prostate cancer cases of Caucasian men, but only 10-30% of prostate cancer cases of African American and Eastern Asian 47-49 patients. As well, less common homozygous loss of PTEN but more common ERF 3 and LASMP alterations were observed in tumors from African American patients than tumors from Caucasian patients 50. Our data informs genomic disparities in prostate cancer between the Sardinian population and other ethnic groups. Compared to TCGA prostate cancer cohort, the Sardinian prostate cancer cohort had a lower rate of ERG fusion but a higher rate of UDP**-**glucuronosyltransferase family amplification on chromosome 4q13.2 **(Figure 1C and Figure 4B)**. In addition, a novel BTBD7-SLC2A5 fusion have been identified and occurred in 13% of the Sardinian patients **(Figure 1C and Figure 4B)**. These observations underscore the importance of the inter-population molecular heterogeneity of prostate cancer.

UDP glucuronosyltransferase family genes catalyze the addition of the hydrophilic moiety, glucuronide, to acceptor molecules in a process called glucuronidation 51-53. In humans there are two major classes of UDP glucuronosyltransferase, UGT1 and UGT2, each of which contains multiple genes on chromosome 2 and 4, respectively. Within the UGT2 genes, UGT2B15 and UGT2B17 have been broadly investigated in prostate cancer because of their ability to inactive DHT and testosterone. It have been reported that their overexpression was associated with increased risk of biochemical recurrence 54, 55. Consistent with those findings, we have observed from the data in The Cancer Genome Atlas prostate cancer that patients with high expression of UGT2B15 have increased risk of biochemical recurrence **(Supplementary Figure S6)**. However, the UGT2 family contains multiple genes and different UGT2 genes may play different roles in tumors. In Sardinian prostate cancers we observed an amplification in chromosome 4q13.2 which contains the UGT2 genes, UGT2B4, UGT2B7, UGT2B10, UGT2B11, UGT2A1, UGT2A2, UGT2B28, and UGT2A3** (Figure 2A&B)**. UGT2B4 was identified as the location of the peak of amplification **(Figure 2A)**. Interestingly, UGT2B4 is noticeable for its ability in the clearance of estrogens 56. It was reported that polymorphisms of UGT2B4 have been associated with increased breast cancer risk 57. To examine the impact of UGT2B4 expression in prostate cancer, we investigated the expression data of pan-cancer study from TCGA. Our analysis showed that the expression level of UGT2B4 in tumors of breast cancer was significantly lower than normal tissues **(Supplementary Figure S3)** while UGT2B4 was significantly higher in tumors than normal tissues of prostate cancer** (Figure 5A)**. Furthermore, we found that high expression of UGT2B4 was associated with low-grade prostate cancer and favorable disease-free survival **(Supplementary Figure S4)**. As well, we found that high expression of UGT2B4 was associated with upregulated tumor metabolic pathways such as 'de novo' IMP biosynthetic process, glutamine and monocarboxylic acid metabolism **(Figure 5D)**. These data suggest that the UGT family may have different roles in cancer development or progression depending on the type of cancer, stage of disease, and type of mutation.

In addition, we did find a novel fusion event, BTBD7-SLC2A5, in 13% of the Sardinian patients, whose breakpoints were within an exon region **(Figure 3A)** and potentially translate a novel functional protein that contains the entire transmembrane domain and fructose transport domain of SLC2A5 and the BTB domain (Broad-Complex, Tramtrack and Bric a brac domain) of BTBD7 **(Figure 3B)**. BTB/POZ domain-containing protein 7 (BTBD7) BTBD7 regulates the dynamics of cell adhesion and motility during epithelial branching morphogenesis 58, and has been reported to be associated with various cancers 59-61. SLC2A5 is a fructose transporter and has been reported to be associated with various cancers as well 62-64. Lung cancers with mutations in SLC2A5 promote lung adenocarcinoma cell growth and metastasis by enhancing fructose utilization 63. BTBD7-SLC2A5 may be involved in unregulated cell adhesion, motility and epithelial morphogenesis due to loss of exons 10 and 11 of BTBD7, or be involved in shifting of energy metabolism to enhance tumor growth due to containing entire transmembrane domain and fructose transport domain of SLC2A5. However, further investigated in the future is needed to determine the importance of this fusion.

Overall, genetic alterations in Sardinian prostate cancer tumors that were identified in our study inform the importance of the inter-population molecular heterogeneity of prostate cancer. Furthermore, understanding their functional mechanism will aid in developing drugs to benefit the prostate cancer patients.

## Conclusion

Our data reveals a low rate of ERG fusion in Sardinian prostate cancer. Interestingly, we identified a novel BTBD7-SLC2A5 fusion that occurred in 13% of the patients and we also found that the UGT2B4 on 4q13.2 was amplified in 20% of the patients. These observations underscore the importance of the inter-population molecular heterogeneity of prostate cancer. In addition, our analysis provides insight into clinical relevance and functional mechanism of UGT2B4 expression in prostate cancer. Further understanding their functional mechanism of action will aid in developing drugs to benefit the prostate cancer patients.

## Supplementary Material

Supplementary figures and tables.

## Figures and Tables

**Table 1:** Clinical parameters of 30 prostate cancer patients

Clinical parameters	Number of Patients
**Gleason Score**	
3+3	6
3+4	13
4+3	9
4+3	1
4+3+5	1
**PSA**	
1~4	4
4~10	20
10~20	6
**Age**	
53~59	4
60~69	20
70~74	6
**Risk Stratification**	
low risk	23
intermediate risk	6
high risk	1
**Survival**	
dead within 5 years	1
dead after 5 years	5
alive	24
ADT Treatment Naive	30

**Table 2:** Identification of BTBD7-SLC2A5 fusion events in Whole exome sequnceing through junction-spanning reads

SampleName	FusionGene	Chr 1	Position 1	Strand 1	Chr 2	Position 2	Strand 2	SplitRead	RescuedSplitRead	UniqueCuttingPosition
306	BTBD7-SLC2A5	14	93712486	-	1	9121449	+	15	0	5
409	BTBD7-SLC2A5	14	93712486	-	1	9121449	+	12	2	6
206	BTBD7-SLC2A5	14	93712486	-	1	9121449	+	15	2	14
815	BTBD7-SLC2A5	14	93712486	-	1	9121449	+	12	1	10

SplitRead: count of seed reads which at least 25nt mapped in both BTBD7 and SLC2A5; RescuedSplitRead: count of rescued reads which at least 15nt mapped in either BTBD7 or SLC2A5; UniqueCuttingPosition: it expected that a real fusion would have more than one unique cutting position.
