# Supplementary material for: Whole-exome Sequencing of Prostate Cancer in Sardinian Identify Recurrent UDP-glucuronosyltransferase Amplifications
Source: J Cancer. 2021 Jan 1;12(2):438–50. doi: 10.7150/jca.48433 (PMC7738997; doi:10.7150/jca.48433)
Supplement: Supplementary file 1 — Supplementary figures and tables. [file jcav12p0438s1.pdf]

Supplementary Information

Supplementary Table and Figure legends

Table S1 Primers for dectection of ERG fusion

Mutation ID	5' Partner Gene			3' Partner Gene			Forward Primer	Reverse Prime
	Gene Name	Last Observed Exon	Inferred Breakpoint	Gene Name	First Observed Exon	Inferred Breakpoint		
COSF125	TMPRSS2	1(utr)	79	ERG	4	312	F:GCTAAGCAGGAGGCGGAGGC	R:CGTAGGCACACTCAAACAACGACTG
COSF128	TMPRSS2	2	150	ERG	4	312	F:GATAACAGCAAGATGGCTTTGAACT	R:TAGGCACACTCAAACAACGACTGGT
COSF123	TMPRSS2	1(utr)	79	ERG	2(utr)	124	F:GCTAAGCAGGAGGCGGAGGC	R:CGTAGGCACACTCAAACAACGACTG
COSF126	TMPRSS2	1(utr)	79	ERG	5	530	F:CTAAGCAGGAGGCGGAGGCGGAGGC	R:GCTGCCCACCATCTTCCCGCCTTTG
COSF127	TMPRSS2	2	150	ERG	2(utr)	124	F:GATAACAGCAAGATGGCTTTGAACT	R:TAGGCACACTCAAACAACGACTGGT

Supplementary Table S2 Comparision of chromosome 4q13.2 amplification rate between Sardinian prostate cancer cohort and the other 13 prostate cancer genomic studies

Projects	Primary or Metastaic tumor	Total cases (n)	Cases with 4q13.2 amplification (n)	Cases In Sarinian Cohort (n)	4q13.2 amplification cases in Sardinian Cohort(n)	Pval（Fisher Test）
PRAD(MSKCC/DFCI2018)	Primay & Metastaic	1013	15	30	6	1.35E-05
Prostate(SU2C2019)	Metastaic	444	6	30	6	2.74E-05
Prostate(TCGA)	Primay	497	1	30	6	1.38E-07
Prostate(SU2C)	Metastaic	150	1	30	6	8.44E-05
Prostate(MSKCC2010)	Primay	240	0	30	6	1.17E-06
Prostate(FHCRC,2016)	Primay	176	0	30	6	6.02E-06
Prostate(MSKCC2014)	Primay	104	0	30	6	8.27E-05
MSK−IMPACT Prostate	Metastatic	424	0	30	6	5.05E-08
Prostate(Broad/Comell2012)	Primay	112	0	30	6	5.80E-05
Prostate(Eur Urol2017)	Primay	65	0	30	6	6.83E-04
Prostate(MICH)	Metastaic	61	3	30	6	5.46E-02
Prostate(Broad/Comell2013)	Metastaic	56	3	30	6	5.98E-02
The MPC Project	Metastaic	19	1	30	6	2.24E-01

Supplementary Figures S1-6 (pdf):

A

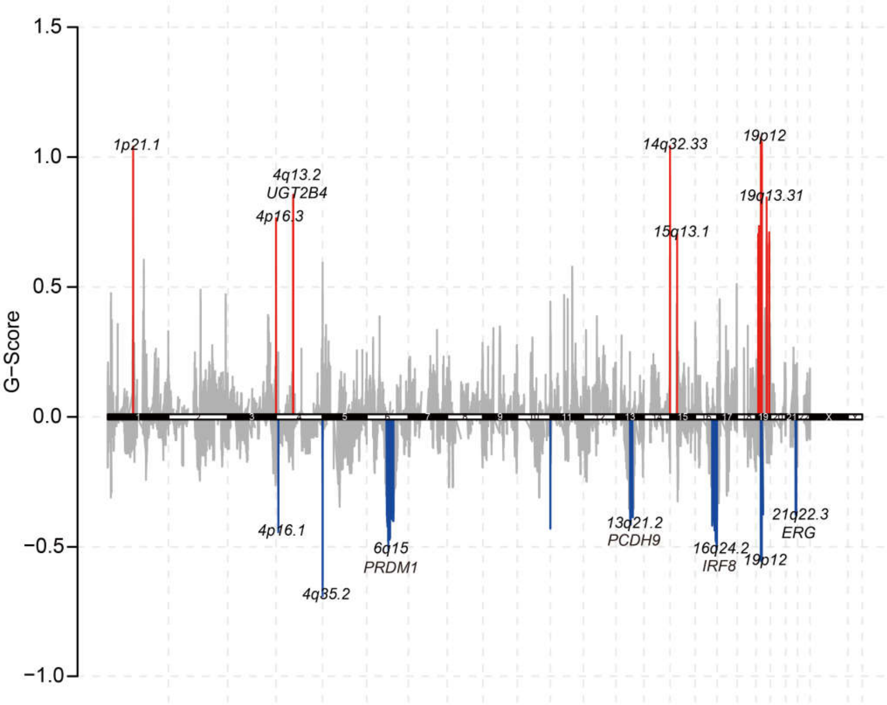

B

cytoband	1p21.1	14q32.33	19p12	19q13.31	4p16.3	4q13.2	15q13.1
q value	2.95E-05	2.95E-05	2.95E-05	4.07E-04	1.85E-03	3.66E-04	4.65E-03
residual q value	2.95E-05	2.95E-05	1.98E-04	4.07E-04	1.85E-03	4.38E-03	4.65E-03
genes in peak	AMY1A, AMY1B	LINC00226	UQCRFS1	CEACAM1, CEACAM8	ZNF595, ZNF718	UGT2B4, UGT2B7	GOLGA8G, GOLGA8F
is oncogene	no	no	no	no	no	steroid synthesis	no

C

cytoband	4q35.2	19p12	6q15	16q24.2	4p16.1	13q21.2	21q22.3	8p23.1
q value	3.15E-06	1.95E-04	4.35E-04	4.30E-04	1.98E-03	3.74E-03	7.99E-03	1.44E-01
residual q value	3.15E-06	1.95E-04	4.35E-04	4.35E-04	1.98E-03	3.74E-03	7.99E-03	1.44E-01
genes in peak	DUX4, DUX2	UQCRFS1	PRDM1	IRF8	DRD5, HMX1	PCDH9, PCDH8	ERG	NKX3-1
is TSG	repeat sequence	no	yes	yes	no	yes	fusion	yes

Figure S1. G-score across the whole exome region of 30 Sardinian prostate cancers. G-scores were calculated by the GISTIC algorithm to determine the amplitude and the frequency of copy number variation.

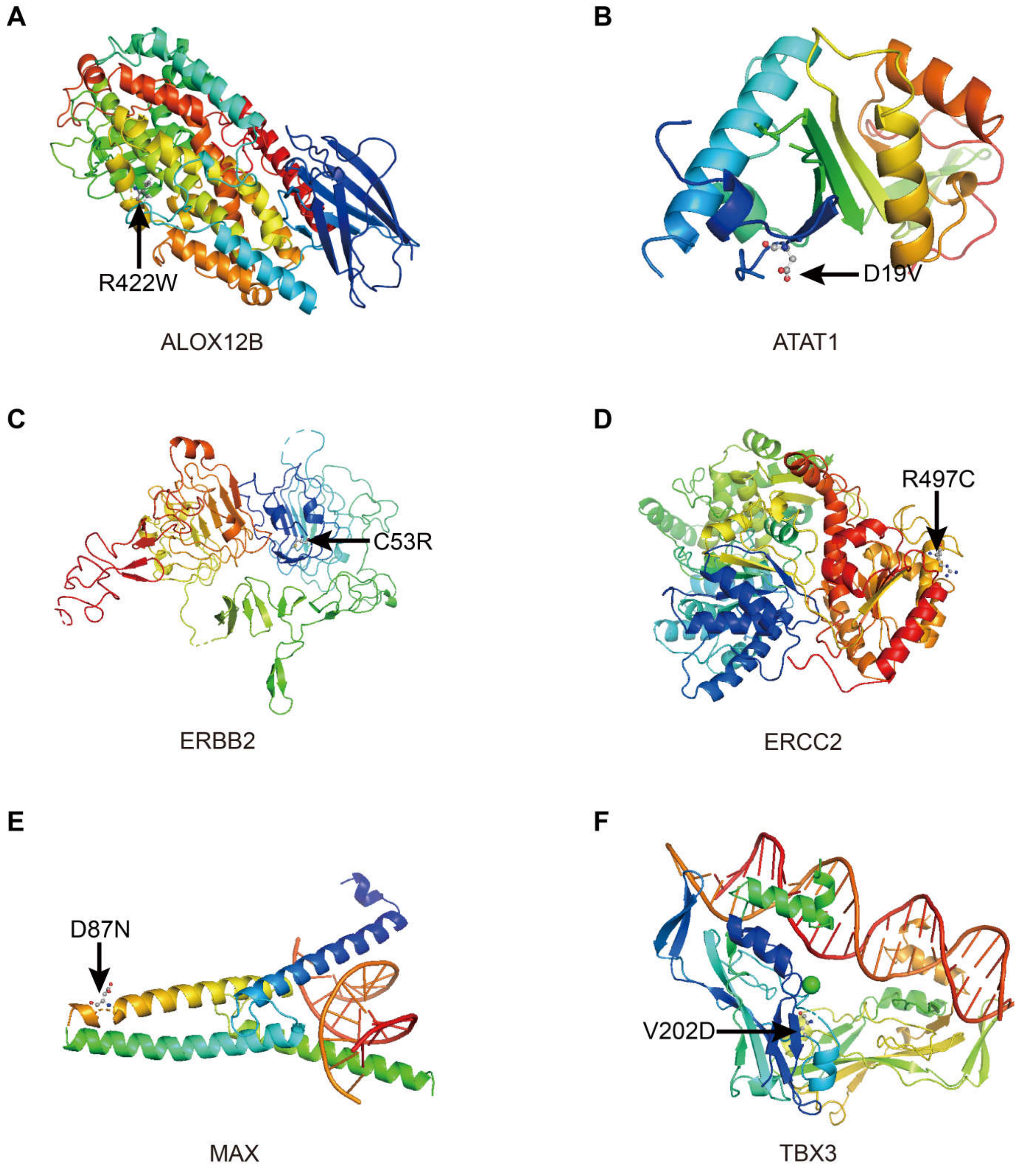

Figure S2. Ribbon structure of Novel candidate driver mutations. (A) R422W mutation in the ALOX12B protein. **(B)** D19V mutation in ATAT1 protein. **(C)** C53R mutation in the ERBB2 Receptor domain. **(D)** R497C mutation in the ERCC2 protein. **(E)** D87N mutation in the MAX protein. **(F)** V202D mutation in the TBX3 T-box domain.

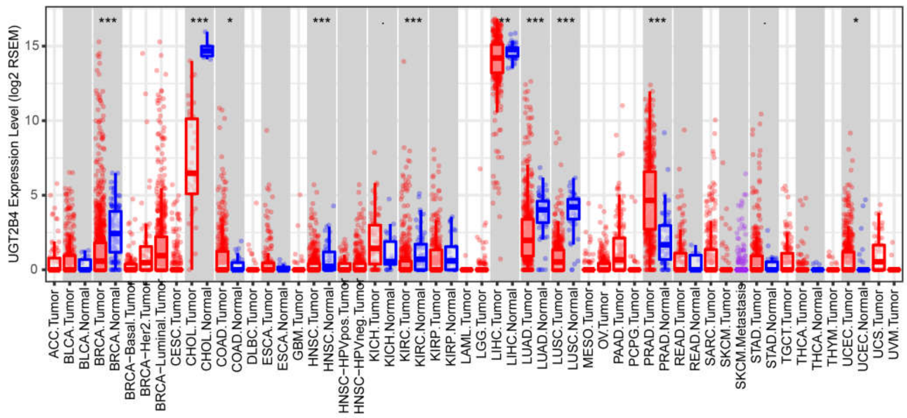

Figure S3. UGT2B4 expression level in pan-cancer. UGT2B4 expression level in TCGA pan-cancer cohort was investigated by https://cistrome.shinyapps.io/timer/. UGT2B4 was significantly down regulated in breast cancer compared to normal tissues but up regulated in prostate tumors compared with normal prostate tissue.

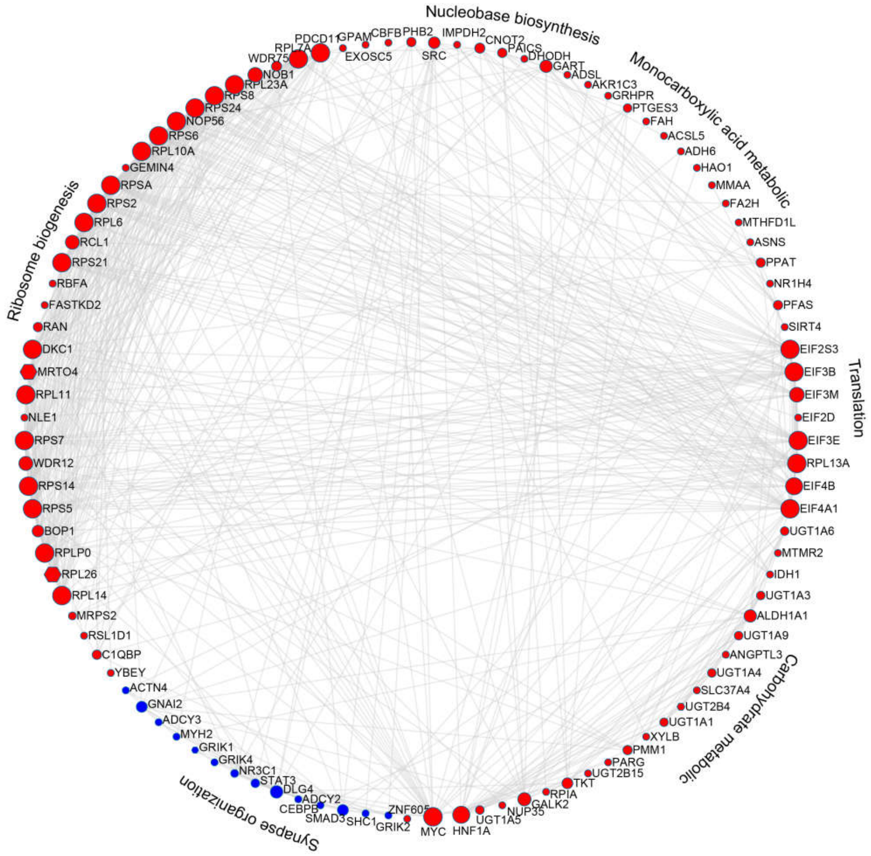

Figure S4. PPI network of UGT2B4 co-expression genes in prostate cancer. Protein-Protein interaction network of UGT2B4 co-expressed genes were downloaded from InWeb_IM (https://www.intomics.com/) and visualized by Cytoscape.

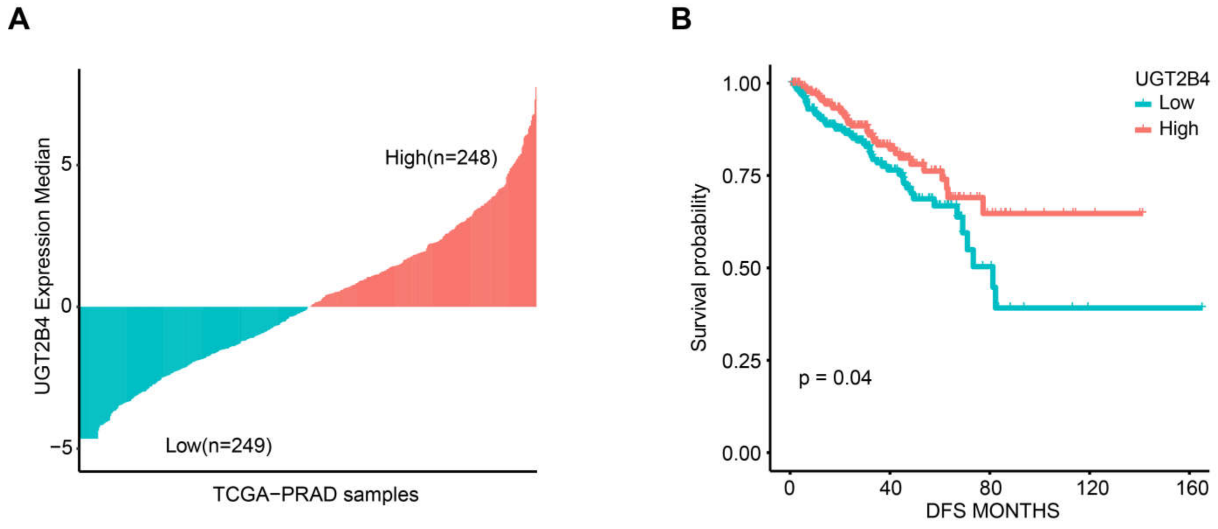

Figure S5. UGT2B4 expression is a favorable prognosis factor. **Patients with high expression of UGT2B4 had longer disease-free survival compared with those patients with low expression of UGT2B4. (A)** All 497 patients from The Cancer Genome Atlas database were grouped by the median UGT2B4 expression level of the cohort. X-axis. **(B)** Kaplan–Meier analysis demonstrated that high UGT2B4 expressers (red) had longer disease-free survival than the low expressers patients (blue)($p<0.05$).

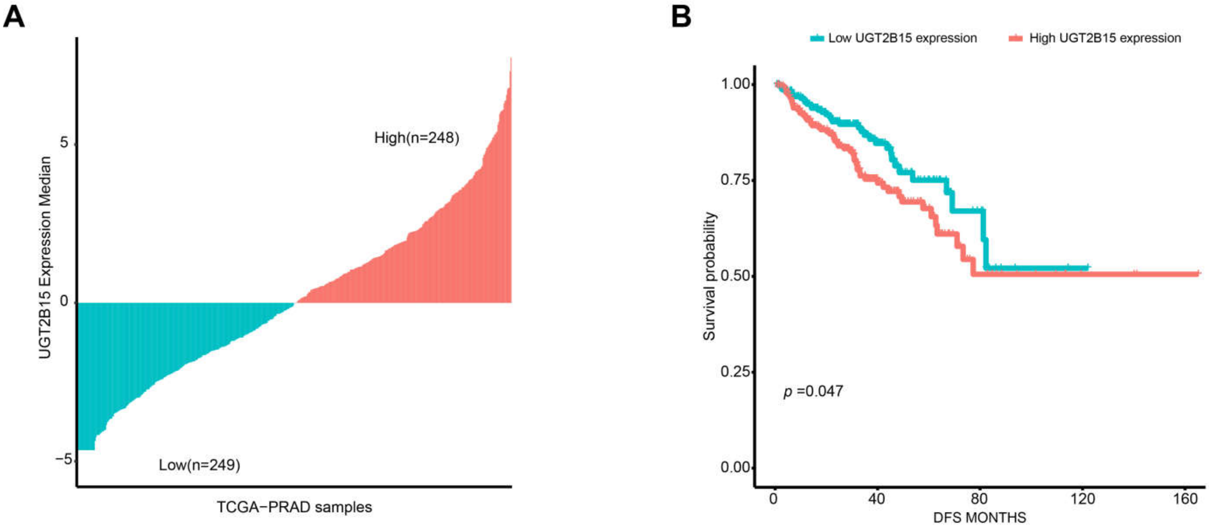

Figure S6. UGT2B15 expression is an adverse prognosis factor**. (A)** All 497 patients from The Cancer Genome Atlas database were grouped by the median UGT2B15 expression level of the cohort. **(B)** Kaplan–Meier analysis demonstrated that high UGT2B15 expressers (red) had shorter disease-free survival than the low expressers patients (blue)($p<0.05$).
